# Drought and recovery in barley: key gene networks and retrotransposon response

**DOI:** 10.3389/fpls.2023.1193284

**Published:** 2023-06-12

**Authors:** Maitry Paul, Jaakko Tanskanen, Marko Jääskeläinen, Wei Chang, Ahan Dalal, Menachem Moshelion, Alan H. Schulman

**Affiliations:** ^1^ HiLIFE Institute of Biotechnology, University of Helsinki, Helsinki, Finland; ^2^ Viikki Plant Science Centre (ViPS), University of Helsinki, Helsinki, Finland; ^3^ Production Systems, Natural Resources Institute Finland (LUKE), Helsinki, Finland; ^4^ The Robert H. Smith Institute of Plant Sciences and Genetics in Agriculture, The Hebrew University of Jerusalem, Rehovot, Israel

**Keywords:** drought, autophagy, barley, resilience (environmental), network analysis, gene expression, *Hordeum vulgare*

## Abstract

**Introduction:**

During drought, plants close their stomata at a critical soil water content (SWC), together with making diverse physiological, developmental, and biochemical responses.

**Methods:**

Using precision-phenotyping lysimeters, we imposed pre-flowering drought on four barley varieties (Arvo, Golden Promise, Hankkija 673, and Morex) and followed their physiological responses. For Golden Promise, we carried out RNA-seq on leaf transcripts before and during drought and during recovery, also examining retrotransposon *BARE*1expression. Transcriptional data were subjected to network analysis.

**Results:**

The varieties differed by their critical SWC (ϴ_crit_), Hankkija 673 responding at the highest and Golden Promise at the lowest. Pathways connected to drought and salinity response were strongly upregulated during drought; pathways connected to growth and development were strongly downregulated. During recovery, growth and development pathways were upregulated; altogether, 117 networked genes involved in ubiquitin-mediated autophagy were downregulated.

**Discussion:**

The differential response to SWC suggests adaptation to distinct rainfall patterns. We identified several strongly differentially expressed genes not earlier associated with drought response in barley. *BARE1* transcription is strongly transcriptionally upregulated by drought and downregulated during recovery unequally between the investigated cultivars. The downregulation of networked autophagy genes suggests a role for autophagy in drought response; its importance to resilience should be further investigated.

## 1 Introduction

Optimization of the balance between carbon fixed and water lost by transpiration is a central problem for plants. In drought, the challenge to water homeostasis from decreased water potential generates a response by the plant at a critical soil water content (SWC), which leads to stomatal closure, even in daylight, to reduce water loss. The associated dehydration and its consequences are commonly referred to as drought stress. Drought response involves mechanisms and signaling cascades, leading to stomatal closure (Merilo et al., 2015) and to physiological and metabolic changes (Jogawat et al., 2021). Mechanisms to counter dehydration and cellular damage include accumulation of osmolytes to mediate osmotic adjustment (Hildebrandt, 2018) and enzymatic and non-enzymatic scavenging of excess reactive oxygen species (ROS) (Das and Roychoudhury, 2014), as well as changes in the chloroplast proteome (Chen et al., 2021a).

Two contrasting responses to water limitation have been described: isohydric and anisohydric (Sade et al., 2012). Isohydric implies maintenance of constant water potential; many physiological parameters are involved (Scharwies and Dinneny, 2019). Barley has been shown to be diurnally anisohydric (Tardieu and Simonneau, 1998). The terms also have been applied broadly for drought response strategy (Sade et al., 2012), isohydric plants conservating water, closing stomata at a relatively high SWC, sacrificing carbon fixation but delaying plant dehydration. This strategy may reduce risk from early droughts in climates where the probability of precipitation increases during the growing season. An anisohydric plant delays stomatal closure and continues fixing carbon, a strategy consistent with environments having terminal droughts or with those where dry periods are short and show little seasonal variation.

Studies in Arabidopsis indicate that induction of autophagy is needed for drought tolerance (Avin-Wittenberg, 2019). Autophagy is an evolutionarily conserved mechanism for recycling damaged proteins and cellular organelles by transport to the vacuoles or lysosomes for degradation (Chen et al., 2021b). Rewatering at the end of drought may result in the resumption of normal diurnal stomatal opening, recovery of photosynthesis, and the resumption of growth. The occurrence, degree, and rate of recovery strongly depends both on the intensity and duration of drought and on the species; recovery has relatively little studied on its own (Xu et al., 2010; Lawas et al., 2019; Chen et al., 2021b; Qi et al., 2021) but involves the ABA signaling pathway (Cao et al., 2021).

Drought is also well known to induce transcription of transposable elements, particularly retrotransposons (RLX; Wicker et al. (2007). The RLXs have been shown to be transcriptionally induced by stresses ranging from ionizing and UV radiation to drought and heavy metals (Kimura et al., 2001; Grandbastien et al., 2005; Ramallo et al., 2008; Makarevitch et al., 2015; Galindo-Gonzalez et al., 2017). For barley (*Hordum vulgare *L.) RLX, earlier studies indicated that *BARE*1 transcripts and translational products accumulated under drought (Jääskeläinen et al., 2013) and appeared linked to the abscisic acid (ABA) signaling pathway through the ABA-response elements (ABREs) present in the *BARE*1promoter (Jääskeläinen et al., 2013).

Optimization of drought response for an annual crop such as barley is related to the climatic zone for which the plant is bred. In some zones, the probability of drought may be greater early in the growing season (e.g., Nordic conditions, before flowering); in others, the probability of drought increases during seed maturation (Mediterranean basin, as terminal drought) (Rollins et al., 2013; Tao et al., 2017). Here, we focus on pre-flowering drought in four barley cultivars shown in pilot experiments to respond differentially to SWC (Dalal et al., in prep). For one of them (Golden Promise), the goal was to carry out transcriptional analyses for leaves for both genes and the *BARE*1 RLX at physiologically defined time points before, during, and after drought. Gene expression data were then input to network analysis to understand the activated pathways and possible connections to RLX stress response.

## 2 Materials and methods

### 2.1 Plant material

Barley cultivars Arvo, Golden Promise (“GP”), Hankkija 673 (“H673”), and Morex were selected from the diversity set of the ERA-NET SusCrop Climbar Project. GP is the standard genotype for transformation in barley (Schreiber et al., 2019), whereas preliminary experiments indicated that Arvo and H673 have high and low stomatal conductance, respectively (Dalal et al., in prep.). Morex is the established reference genome assembly in barley (Beier et al., 2017; Mascher et al., 2017; Mascher et al., 2021).

### 2.2 Growth conditions

**Figure 1:**
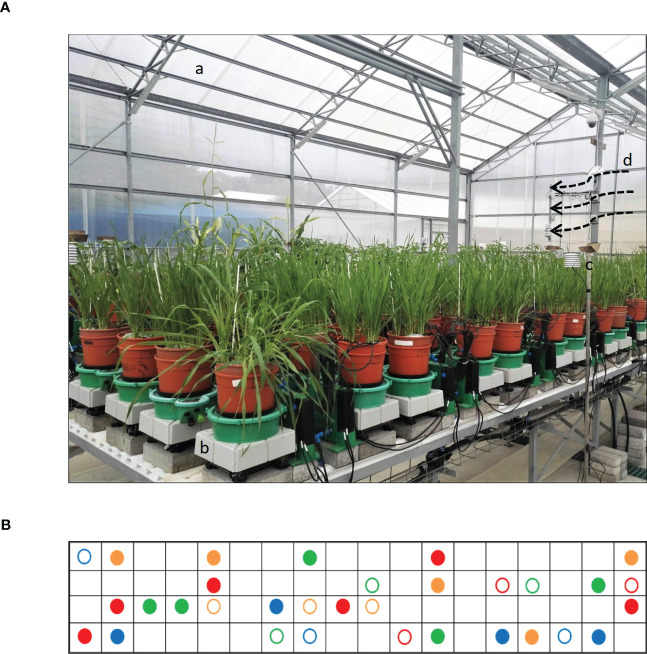
Experimental setup. **(A)** Lysimeters of gravimetric functional phenotyping platform, comprising 72 lysimeter platforms with soil probes and four weather stations. The setup features: (a) minimally controlled greenhouse, (b) lysimeter platform, (c) weather station, and (d) cooling vent. **(B)** Table diagram for sample pots distributed according to a randomized block design. Filled circles are drought treatments; empty circles are well-watered (controls). Arvo, orange; GP, red; H673, green; Morex, blue.

Plants were grown during the winter season in the iCORE functional phenotyping greenhouse (https://plantscience.agri.huji.ac.il/icore-center ) under natural sunlight, with moderate temperature control, which closely reflects the natural external environment (Halperin et al., 2017; Galkin et al., 2018) (**Figure 1A**). There, drought experiments were carried out with a randomized block design on a gravimetric functional phenotyping platform (Dalal et al., 2020; Gosa SC et al, 2022) (Plantarray, PA 3.0, PlantDitech Ltd., Yavne, Israel), which allows simultaneous standardized control of drought treatment with continuous monitoring of multiple physiological parameters. The platform comprises temperature-compensated load cells (lysimeters) for simultaneous and continuous gravimetric monitoring of water relations in the soil–plant–atmosphere continuum (SPAC) under dynamic environmental conditions (Halperin et al., 2017).

**Figure 2:**
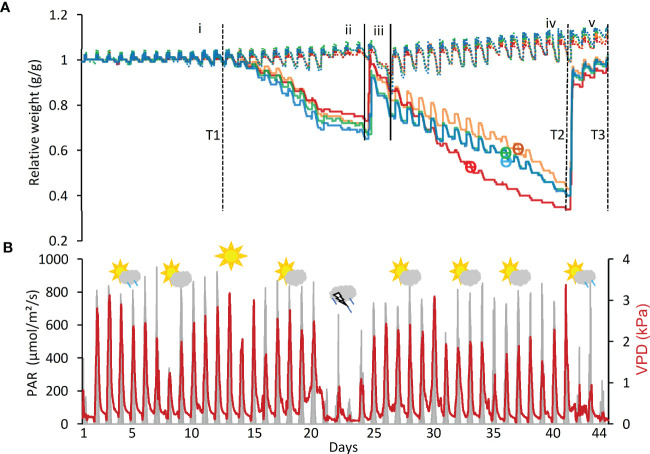
Key system parameters during drought and recovery. **(A) **Relative change in system weight of all control and drought-treated pots. The experiment is divided into five phases:(i)well-watered,(ii)dry phase I,rewatering I, (iv) dry phase II, and (v) rewatering II. Drought samples, solid lines; well-irrigated controls, dashed. Cultivars are Arvo, orange; Golden Promise (GP), red; Hankkija 673 (H673), green; Morex, blue. Day of ϴ crit indicated by ϴ on the cultivars’ curves. Time points (T) for collection of RNA from leaf samples are: T1, last day of well-watered; T2, last day of drought; T3, last day of recovery. **(B)** Photosynthetically active radiation (PAR) and vapor pressure deficit (VPD) over the course of the experiment. The weather symbols depict conditions outdoors during this time each symbol representing the average of five days.

Seeds were sown in germination trays and then transplanted at the 3- to 4-leaf stage (2 weeks old) to pots. Before transplanting, seedling roots were carefully washed to remove the original soil, planted into potting soil [“Bental 11”, Tuff Marom Golan, Israel; four per 3.9-liter pot; Dalal et al. (2020)], and then placed onto lysimeters (Dalal et al., 2019; Gosa SC et al, 2022). Each cultivar was grown in four to six biological replicates for the drought treatment and in three biological replicates for the controls, which were maintained under well-irrigated conditions (**Figure 1B**). The plants were grown at 18°C–25°C, 20%–35% relative humidity, and 10-h light/14-h dark, typical of the greenhouse’s semi-controlled conditions as affected by the external Mediterranean winter. Temperature, humidity, and photosynthetically active radiation (PAR) were monitored as described earlier (Dalal et al., 2020; Gosa SC et al, 2022). The vapor pressure deficit (VPD) ranged between 0.2 and 4.0 kPa in the greenhouse and represented typical weather fluctuations during winters in Central Israel (**Figure 2B**).

### 2.3 Experimental design

**Figure 3:**
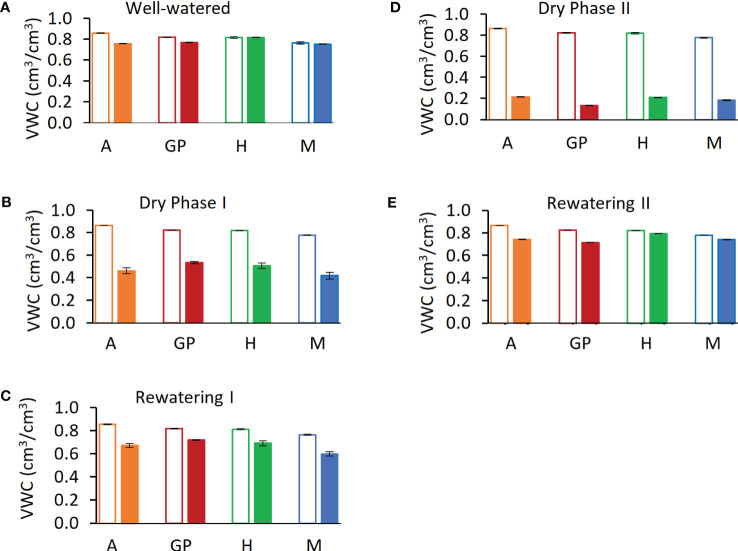
Calculated soil volumetric water content (VWC) during five phases of the experiment. **(A)** Day 12, last day of well-watered (phase i). **(B)** Day 24, last day of dry phase I **(C)** Day 25, last day of rewatering I **(D)** Day 41, dry phase II. **(E)** Day 44, last day of rewatering II. Solid bars represent drought samples; empty bars represent controls. Arvo (A), orange; red, Golden Promise (GP), green; Hankkija 673 (H); blue, Morex (M)..

**Figure 4:**
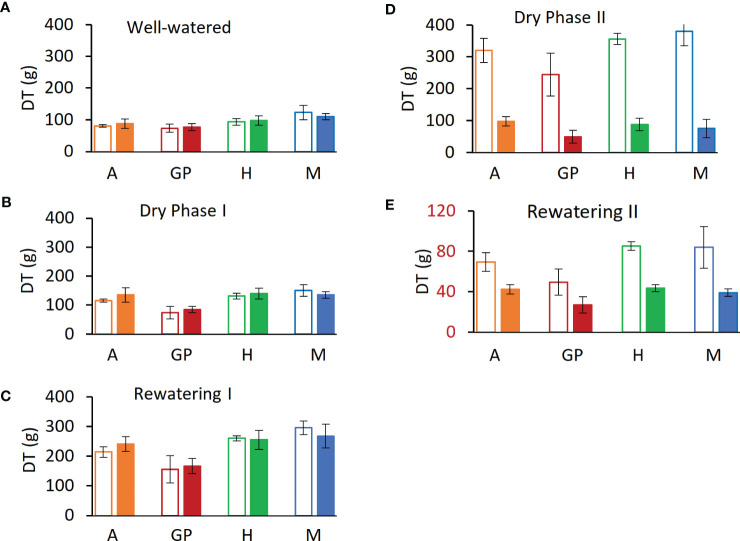
Daily transpiration (DT) at the end of each experimental phase. **(A)** Day 12, last day of well-watered (phase i). **(B)** Day 24, last day of dry phase I **(C)** Day 25, last day of Rewatering I **(D)** Day 41, dry phase II. **(E)** Day 44, last day of rewatering II. Solid bars represent drought samples, empty bars controls. Orange represents Arvo (A); red, Golden Promise (GP); green, Hankkija 673 (H); blue, Morex (M).

To mimic the onset of a drought, the experiment was divided into five phases on the lysimeter platform: (i) well watered, (ii) dry phase I (iii), rewatering I (iv), dry phase II, and (v) rewatering II (**Figure 2A**). For the well-watered phase (first 12 days) and for the controls throughout, plants daily received three nighttime irrigations at 3-h intervals to reach field capacity. The volumetric water content (VWC) was calculated with the SPAC analytical software embedded in the Plantarray system (**Figure 3**). In dry phase I, the system was set to irrigate droughted plants to 80% of their own previous day’s transpiration, so that these would be subjected to gradual water stress from days 13 to 24, followed by rewatering (rewatering I) on the night of day 25. Dry phase II extended from days 26 to 41 with the same irrigation parameters as dry phase I, until the time when the droughted plants reached <100 g of daily transpiration (DT; around 20% to 30% of controls) (**Figure 4D**). Rewatering II began on day 42. Daily night irrigation was given to all plants until day 44. At that point, several irrigation cycles were used to moisten the soil and to ensure a laterally uniform SWC in the pots. VPD data were continuously monitored (**Figure 2B**).

### 2.4 Sampling and isolation of RNA

Second leaves from the youngest tillers were collected for RNA analysis: time point 1 (T1) on the last day of the well-watered phase (day 12); T2, on the last day of dry phase II (day 41); T3, last day of rewatering II (day 44). Well-watered control samples at the same growth stages were collected at T2 and T3. For RNA extraction, leaf samples were collected in liquid N2 and later ground and stored in TRIzol reagent (Invitrogen). Total RNA was extracted from the leaf samples with the Direct-zol RNA Miniprep Plus kit according to the manufacturer’s (Zymo Research Europe GmbH) guidelines. RNA (1 µg) from each sample was treated with Ambion® DNase I (RNase-free; Applied Biosystems) in a 20-µl reaction mixture for 90 min at 37°C to remove remaining DNA. An RNeasy MinElute Cleanup Kit (Qiagen) was used to purify and concentrate the RNA from the DNase treatments. RNA quantity and quality were checked both spectrophotometrically (NANODROP-2000 spectrophotometer, Thermo Scientific) and by agarose gel electrophoresis.

### 2.5 qPCR analysis

Reverse transcription to cDNA was carried out in reactions containing 200 ng of total RNA, 1 µl of Invitrogen™ SuperScript® IV Reverse Transcriptase (RT) (Thermo Fisher Scientific), 1 µl of 10 mM deoxynucleotide triphosphates (dNTPs; Thermo Scientific),, and 0.5 µl of 10 µM primer E1820 (AAGCAGTGGTAACAACGCAGAGTACT30NA) as the oligo(dT) primer (Chang et al., 2013). For qPCR, 10 µl of reactions comprised 0.5 µl of cDNA as template, 5 µl of SsoAdvanced™ Universal SYBR® Green Supermix, 0.1 µl of each forward and reverse primer, and 4.3 µl of water. Reactions were run on a CFX 384 Real-Time PCR detection system (Bio-Rad) in three technical replicates as follows: activation for 2 min at 94°C; 40 cycles comprising denaturation for 30 s at 94°C, annealing for 30 s at 56°C, and extension for 1 min at 72°C. Relative quantification of the cDNA product was made by the 2**− ΔΔCT** method (Livak and Schmittgen, 2001). All primers used for qPCR are listed in **Supplementary Table S1**. The amplification efficiency of all the primers has been checked; R2 was from 0.97 to 0.99.

### 2.6 RNA-seq and network analysis

Three sets of biological replicates from each time point and their corresponding controls were taken from cv. GP for RNA-seq analysis. Libraries were constructed using the TruSeq Stranded Total RNA kit with plant cytoplasmic and chloroplast ribosomal RNA removal (Illumina); adaptors were TruSeq adaptors. FastQC (Andrews, 2010) was used to check the quality control metrics of the raw reads and to trim the RNA-seq data. Adapter sequences were removed with Trimmomatic (Bolger et al., 2014). The STAR alignment method (Dobin et al., 2013) served to map sequence reads to the GP genome (GPV1.48, Ensembl Plants). Next, to quantify transcript expression, the Salmon tool was applied (Patro et al., 2017). Differentially expressed genes (DEGs) and principal component analysis (PCA) plots were obtained with DESeq2 (Love et al., 2014; Hong et al., 2020). Multidimensional scaling (MDS) plots were used to verify the approximate expression differences between sample replicates.

From the clean reads, those with pADJ value < 0.05 for their fold change were run in g:Profiler for functional enrichment analysis and to obtain a Gene Matrix Transposed (GMT) file for Cytoscape (Reimand et al., 2019). Cytoscape_v3.8.2 was then used to develop network clusters for biological pathways. The parameters used were false discovery rate (FDR) q-value cutoff = 1, node cutoff = 0.5, edge cutoff = 0.375, and significance threshold for functional enrichment *p*≤ 0.01.

## 3 Results

### 3.1 Whole-plant water relations over the course of drought and recovery

Four barley varieties (Arvo, GP, H673, and Morex), which, in preliminary experiments, differed in their water use strategies, were placed on lysimeters and measured through the four experimental phases. Transpiration and plant weight were followed continuously. The SWC at which the transpiration rate drops in response to drought [ϴ critical point (ϴ_crit_)] was calculated from the data.

**Figure 5:**
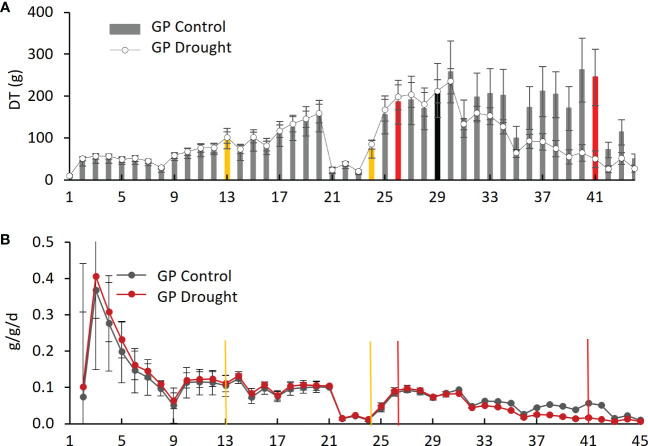
Daily transpiration and plant weight over the experimental period for Golden Promise (GP). **(A)** Daily transpiration (DT). The black bar represents the divergence of drought from control plants. **(B)** Daily specific change in plant weight (g/g/d). Yellow bars represent the start and end of dry phase I, and red bars show the start and end of dry phase II.

*Daily transpiration.*Analysis of DT of the four varieties on the Plantarray platform revealed variation in their transpiration both between the varieties (**Figure 4**, **Supplementary Figure S1**) and day by day (**Figure 5A**, **Supplementary Figures S1A–C**). All plants were exposed to similar changes in PAR and VPD simultaneously (**Figure 1**), which were monitored throughout the experiment (**Figure 2B**); from days 21 to 23, the cloudy and rainy weather outside the minimally controlled greenhouse reduced the PAR and VPD within, lowering DT. For the well-watered control plants of all varieties, DT gradually increased along with plant size over the course of the experiment, closely matched by the droughted plants, which then diverged following the onset of dry phase II. The overall DT of Morex is the highest, followed by H673, Arvo, and GP. The DT of droughted Morex plants diverged from controls by day 20 (**Supplementary Figure S1C**) and H673 by day 25 (**Supplementary Figure S1B**), whereas Arvo and GP only by day 30 (**Figure 5A**, **Supplementary Figure S1A**). A complete return to control whole-plant transpiration was not observed for any of the varieties before termination of the experiment on day 44; all plants showed low DT between days 42 and 44, linked to low VPD and PAR.

### 3.2 Effect of drought on the plant growth and transpiration

Changes in net plant weight as a measure of growth were calculated from the Plantarray system as before (Halperin et al., 2017). The high rate of growth observed for all varieties between days 2 and 9 (**Figure 5B**, **Supplementary Figures S2A–C**) corresponds to the peak in PAR during the same period (**Figure 2B**). The control plants showed similar trends in growth rates throughout the experiment. The droughted plants of all four varieties displayed specific growth rates lower than their well-watered controls during drought: first in Morex (day 23), then Arvo (day 29), and then both H673 and GP (on day 30) (**Supplementary Figure S2**, **Figure 5B**). The relative effect of drought on the growth rate of the four varieties is most apparent when the rate of change is considered.

**Figure 6:**
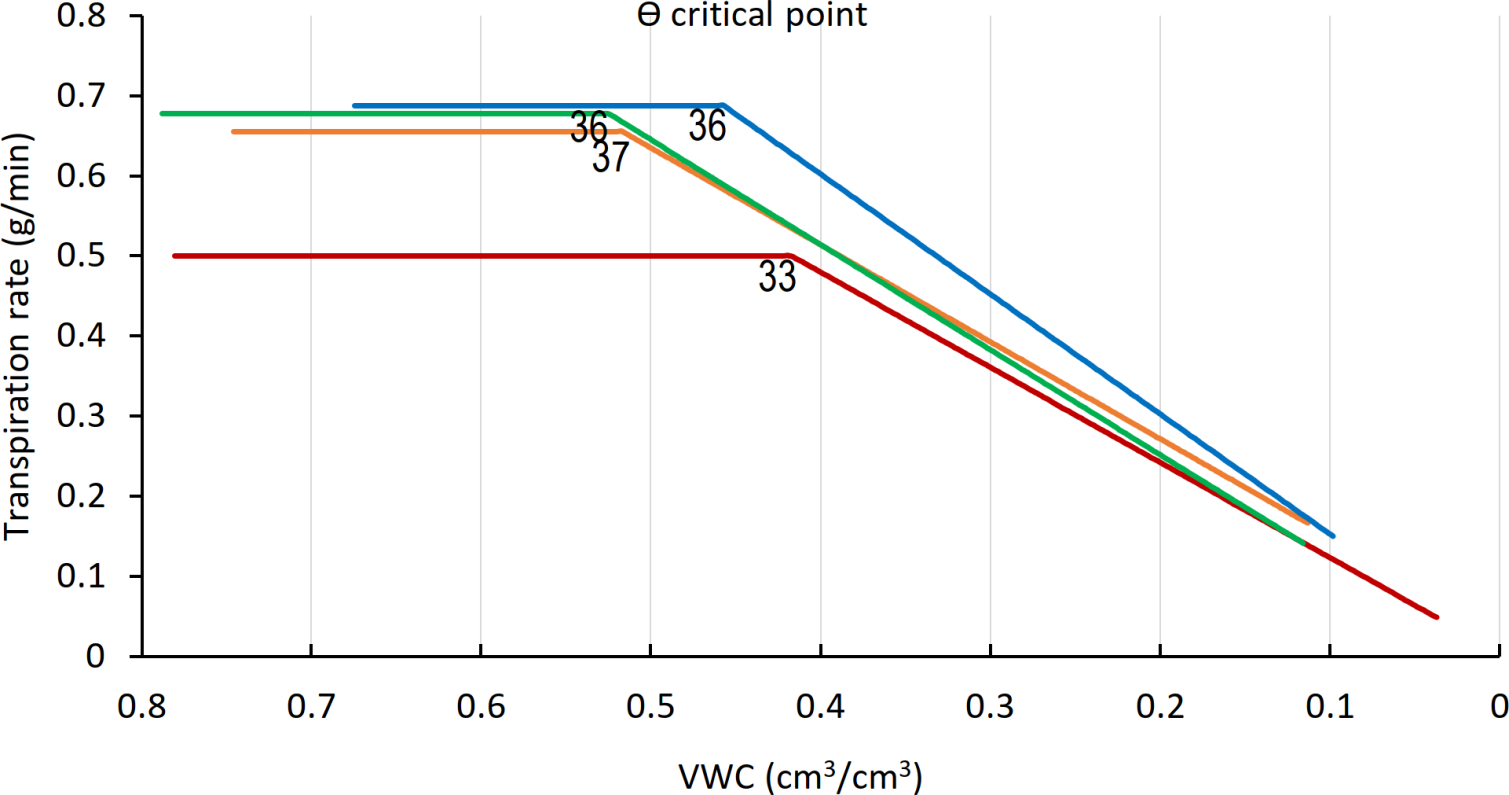
ϴ critical point (ϴ_crit_), for midday whole-plant transpiration versus calculated VWC. The inflection points in the fitted transpiration lines indicate the ϴ_crit_. Arvo, orange; Golden Promise, red; Hankkija 673 (H673), green; Morex, blue. Day on which the ϴ_crit_ occurred is indicated by a number adjacent to the inflection point..

The ϴ_crit_ was calculated for all varieties (**Figure 6**) on the basis of midday whole-plant transpiration (Halperin et al., 2017). The transpiration rates of all four varieties remained constant up to a VWC of 0.55 and then declined, with the VWC at the ϴ_crit_ differing by variety. Arvo, H673, and Morex display similar initial transpiration rates, whereas GP was about 30% lower. The H673 ϴ_crit_ occurred at 0.53 VWC on day 36, Arvo at 0.52 on day 37, Morex at 0.46 on day 36, and GP at 0.42 already on day 33. Following stomatal closure at ϴ_crit_, weight (water) loss preceded at different rates among the varieties. Although GP had the lowest initial transpiration rate (**Figure 6**), it declined the fastest, whereas Arvo, with a high transpiration rate, declined the slowest.

### 3.3 Gene expression analysis

An average of 33 million reads per sample were generated by RNA-seq, which showed the expression of approximately 27,000 genes. To check the quality of the gene expression, DESeq2 dispersion estimates were made to reflect the variance in gene expression for a given mean value; values were adjusted as the basis for significance testing (**Supplementary Figure S3**). PCA of differential gene expression showed an alignment of all groups of samples along PC1 and a narrow dispersion of all control groups along PC2 (**Supplementary Figure S4**). Movement of the expression pattern during the course of the experiment shows a shift within PC2 during drought and then a return to near the position of the control groups for the recovery samples (**Supplementary Figure S4**), consistent with ongoing physiological recovery. Of the genes detected, those differentially expressed (DEGs) with adjusted *p* < 0.01 include 611 that were upregulated and 643 downregulated in drought compared to the well-watered controls samples taken on the same day, whereas 697 were upgregulated and 282 downregulated during recovery compared with the controls on the same day (**Supplementary Tables S2**–**S5**).

RNA-seq reads were annotated by reference to the cv. Morex genome (release 2), transcript expression was quantified, and DEGs were identified. In the drought samples, the most highly upregulated gene compared to the well-watered control was for glutathione s-transferase (**Table 1**), with 3-million-fold more expression than control; in the samples from plants undergoing recovery, this gene dropped by 8.6-fold compared with drought. Altogether, 44 genes showed more than 100-fold upregulation and 218 genes over 10-fold upregulation compared to controls (**Supplementary Table S2**). The most downregulated gene was for a cytochrome P450 family protein (**Table 2**), with around 3,000-fold less expression than control. Conversely, among the DEGs in recovery compared to drought, this gene showed the third highest upregulation, 195-fold (**Supplementary Table S7**). Altogether, nine genes showed more than 100-fold downregulation and 53 genes over 10-fold downregulation (**Supplementary Table S3**).

**Table 1:** Top 20 upregulated genes, drought phase.

Ensembl_gene_id	Description	log2 FC^1^	padj^2^
HORVU.MOREX.r2.5HG0441700	Glutathione s-transferase	21.49	5.6E-07
HORVU.MOREX.r2.6HG0456270	Linalool synthase	13.20	9.4E-05
HORVU.MOREX.r2.6HG0516720	Dehydrin	11.76	9.4E-18
HORVU.MOREX.r2.5HG0395660	U-box domain containing protein	10.67	6.2E-03
HORVU.MOREX.r2.5HG0429440	Dehydrin	10.31	3.4E-05
HORVU.MOREX.r2HG0053260	NOT FOUND	10.17	2.1E-03
HORVU.MOREX.r2.3HG0242240	Coiled-coil domain-containing protein 18	9.89	7.6E-12
HORVU.MOREX.r2.3HG0184850	Trypsin inhibitor	9.44	5.5E-09
HORVU.MOREX.r2.4HG0328360	Branched-chain amino acid aminotransferase	9.38	5.6E-04
HORVU.MOREX.r2.3HG0186590	Basic 7S globulin 2	9.28	2.1E-04
HORVU.MOREX.r2.7HG0593880	Cysteine-rich/transmembrane domain A–like protein	9.19	2.3E-03
HORVU.MOREX.r2.3HG0201230	DWNN domain	9.13	1.9E-04
HORVU.MOREX.r2.7HG0620080	Carbonic anhydrase	9.00	2.7E-03
HORVU.MOREX.r2.2HG0149830	Caleosin	8.39	4.3E-05
HORVU.MOREX.r2.3HG0184880	Trypsin inhibitor	8.38	6.9E-11
HORVU.MOREX.r2.3HG0245240	Disulfide isomerase	8.35	4.6E-07
HORVU.MOREX.r2.5HG0356770	Germin-like protein	8.09	8.1E-04
HORVU.MOREX.r2.5HG0402770	Basic helix-loop-helix (bHLH) DNA-binding superfamily protein	8.04	2.7E-05
HORVU.MOREX.r2.2HG0149820	Caleosin	7.75	9.0E-03
HORVU.MOREX.r2.6HG0512380	2-oxoglutarate (2OG) and Fe (II)-dependent oxygenase superfamily protein	7.61	1.7E-03

^1^FC, fold change; ^2^padj, adjusted *p*-value.

**Table 2:** Top 20 downregulated genes, drought phase.

Ensembl_gene_id	Description	log2 FC1	padj2
HORVU.MOREX.r2.3HG0191820	Cytochrome P450 family protein	−11.71	6.0E-08
HORVU.MOREX.r2.3HG0268430	Basic helix-loop-helix (BHLH) family transcription factor	−9.82	3.1E-03
HORVU.MOREX.r2.4HG0346330	Cortical cell-delineating protein	−9.46	2.6E-09
HORVU.MOREX.r2.4HG0346340	Cortical cell-delineating protein	−9.28	4.7E-08
HORVU.MOREX.r2.2HG0158510	Bifunctional inhibitor/lipid-transfer protein/seed storage 2S albumin superfamily protein	−8.79	4.5E-05
HORVU.MOREX.r2.4HG0288340	translation initiation factor	−7.73	4.0E-07
HORVU.MOREX.r2.7HG0561840	MYB-related transcription factor	−7.69	4.0E-09
HORVU.MOREX.r2.5HG0369380	Glutaredoxin family protein	−7.29	1.0E-04
HORVU.MOREX.r2.3HG0268450	Basic helix-loop-helix (bHLH) DNA-binding superfamily protein	−6.98	2.3E-03
HORVU.MOREX.r2.7HG0548630	Pollen Ole e 1 allergen/extensin	−6.56	7.0E-09
HORVU.MOREX.r2.5HG0356760	Glutaredoxin family protein	−6.47	2.6E-03
HORVU.MOREX.r2.5HG0412710	CRT-binding factor	−6.45	1.5E-04
HORVU.MOREX.r2.2HG0165830	Pollen Ole e 1 allergen/extensin	−6.39	1.3E-09
HORVU.MOREX.r2.4HG0330890	Beta-xylosidase	−6.20	1.6E-03
HORVU.MOREX.r2.3HG0268330	Dicer-like 3	−5.83	4.2E-07
HORVU.MOREX.r2.2HG0079590	Avr9/Cf-9 rapidly elicited protein	−5.72	2.9E-03
HORVU.MOREX.r2.7HG0623060	Aquaporin	−5.61	4.4E-03
HORVU.MOREX.r2.6HG0455680	Pollen Ole e 1 allergen/extensin	−5.57	1.1E-07
HORVU.MOREX.r2.5HG0438030	Leucine-rich repeat receptor-like protein kinase family protein	−5.47	1.3E-05
HORVU.MOREX.r2.2HG0160110	Orexin receptor type 2	−5.39	1.0E-07

^1^FC, fold change; ^2^padj, adjusted p-sub>value.

In the recovery samples, compared to the controls that never experienced drought, the most upregulated gene was annotated as a leguminosin group485 secreted peptide, having 16-billion-fold more expression than in the controls (**Table 3**). This gene has been reported in *Medicago truncatula* root during root nodulation, with gene ID Medtr5g064530 and Medtr2g009450. However, a BLASTp of the transcript corresponding to the barley gene (HORVU.MOREX.r2.5HG0428280.1) has its best match (89% identity, Expected value (*E*-value) of 2e-36) to a serine/threonine-protein phosphatase 7 (PP7) long-form homolog for barley (XP_044965344.1). Four genes showed more than 100-fold upregulation and 58 genes over 10-fold upregulation (**Supplementary Table S4**). Compared to the drought samples, 139 genes showed more than 10-fold upregulation. The most upregulated, compared with drought (635X; **Supplementary Table S7**), was annotated as for a “bifunctional inhibitor/lipid-transfer protein/seed storage 2S albumin superfamily protein,” a protein type reported to have diverse functions (Wei and Zhong, 2014).

**Table 3:** Top 20 upregulated genes during the recovery phase.

Ensembl_gene_id	Description	log2 FC^1^	padj^2^
HORVU.MOREX.r2.5HG0428280	Leguminosin group485 secreted peptide	33.95	5.4E-10
HORVU.MOREX.r2.5HG0406650	Nucleolar-like protein	7.59	2.4E-03
HORVU.MOREX.r2.7HG0563500	Fasciclin-like arabinogalactan protein	7.11	2.7E-04
HORVU.MOREX.r2HG0002090	Arginine decarboxylase	6.73	3.9E-03
HORVU.MOREX.r2.7HG0604490	Xyloglucan endotransglucosylase/hydrolase	6.25	3.4E-03
HORVU.MOREX.r2.2HG0083580	Cytochrome P450	5.57	9.3E-03
HORVU.MOREX.r2.4HG0340230	translation initiation factor	5.54	1.1E-03
HORVU.MOREX.r2.2HG0179370	Cytochrome P450	5.51	1.0E-02
HORVU.MOREX.r2.7HG0579230	MLG (mixed-linkage glucan) synthase, Biosynthesis of MLG (cell wall polysaccharide	5.33	6.3E-04
HORVU.MOREX.r2HG0072930	Thiamine-phosphate synthase	5.05	6.7E-15
HORVU.MOREX.r2.6HG0519350	Heparanase	5.05	2.2E-04
HORVU.MOREX.r2.2HG0141350	3-Oxoacyl-[acyl-carrier-protein] synthase	4.86	4.4E-08
HORVU.MOREX.r2.3HG0183950	Glycosyltransferase	4.81	2.5E-03
HORVU.MOREX.r2.5HG0355750	Bidirectional sugar transporter SWEET	4.80	3.5E-05
HORVU.MOREX.r2.7HG0558300	Glycosyltransferase	4.78	4.4E-03
HORVU.MOREX.r2.5HG0349900	Receptor-like protein kinase	4.67	2.4E-05
HORVU.MOREX.r2.6HG0516480	F-box protein SKIP8	4.52	1.5E-13
HORVU.MOREX.r2.4HG0318550	MYB transcription factor	4.44	5.4E-03
HORVU.MOREX.r2.6HG0502520	4-coumarate: coenzyme A ligase, Flavonoid biosynthesis	4.39	7.9E-03
HORVU.MOREX.r2.7HG0594280	Beta-galactosidase	4.33	6.7E-03

^1^FC, fold change; ^2^padj, adjusted p-value.

The most downregulated transcript in recovery compared to control corresponded to a superfamily *Copia* RLX, which automatic annotation designated as a “retrovirus-related Pol polyprotein”, having around 8-million-fold less expression than the control (**Table 4**). When drought and recovery DEGs were compared, the most strongly downregulated (1.8-million fold) transcript likewise was annotated as a “Pol polyprotein from transposon *Tnt*1-94”, i.e., a superfamily *Copia* RLX (**Supplementary Table S6**). Of the top 20 repressed in recovery compared with drought samples (**Supplementary Table S6**), three were annotated as dehydrins (435- to 1634-fold downregulated) and three as heat-shock proteins (188- to 627-fold downregulated). Compared to controls, overall, three genes showed more than 100-fold downregulation and nine genes more than 10-fold downregulation (**Supplementary Table S5**). Compared to drought samples, 222 genes in the recovery phase showed more than 10-fold downregulation and 140 showed more than 10-fold upregulation. To place the very many significantly DEGs into a biological context, we undertook a network analysis, as described below.

**Table 4:** Top 20 downregulated genes during the recovery phase.

Ensembl_gene_id	Description	log2 FC^1^	padj^2^
HORVU.MOREX.r2.7HG0531380	Retrovirus-related Pol polyprotein from transposon TNT 1-94	−23.01	1.188E-10
HORVU.MOREX.r2.4HG0321820	Plant protein 1589 of Uncharacterized protein function	−7.39	2.631E-05
HORVU.MOREX.r2.3HG0253380	ATP-dependent Clp protease adapter protein ClpS	−6.74	9.786E-03
HORVU.MOREX.r2.4HG0280100	Staurosporin and temperature sensitive 3-like A	−4.50	1.056E-04
HORVU.MOREX.r2.3HG0188940	AP2/B3 transcription factor family protein	−4.31	2.280E-04
HORVU.MOREX.r2.2HG0093560	Flowering-promoting factor 1-like protein 1	−3.70	3.800E-03
HORVU.MOREX.r2.4HG0280110	Staurosporin and temperature sensitive 3-like A/Haloacid dehalogenase-like hydrolase	−3.68	8.266E-05
HORVU.MOREX.r2.7HG0581730	NAC domain-containing protein	−3.64	1.844E-03
HORVU.MOREX.r2.2HG0138540	DNA helicase homolog	−3.51	6.905E-03
HORVU.MOREX.r2.5HG0357350	2-Oxoglutarate and Fe (II)-dependent oxygenase superfamily protein	−3.04	7.989E-03
HORVU.MOREX.r2.2HG0093550	Flowering-promoting factor 1–like protein 1	−2.89	6.611E-04
HORVU.MOREX.r2.2HG0173810	Unidentified	−2.87	2.376E-03
HORVU.MOREX.r2.4HG0279680	Lysine–tRNA ligase	−2.85	1.142E-03
HORVU.MOREX.r2.2HG0091470	NAC domain protein	−2.83	1.016E-04
HORVU.MOREX.r2.6HG0522770	Inosine-5′-monophosphate dehydrogenase	−2.82	6.337E-04
HORVU.MOREX.r2.3HG0259320	Transmembrane protein	−2.76	1.878E-06
HORVU.MOREX.r2.4HG0346330	Cortical cell-delineating protein	−2.68	3.434E-03
HORVU.MOREX.r2.2HG0152820	Hsp70-Hsp90 organizing protein 1	−2.53	1.247E-03
HORVU.MOREX.r2.2HG0133410	SNF1-related protein kinase regulatory subunit gamma 1	−2.52	1.255E-03
HORVU.MOREX.r2.5HG0357520	NAC domain-containing protein/Tetratricopeptide repeat (TPR)-like superfamily protein (LC)	−2.51	2.664E-04

^1^FC, fold change; ^2^padj, adjusted p-value.

### 3.4 Network analysis

#### 3.4.1 Upregulated networks under drought stress

**Figure 7:**
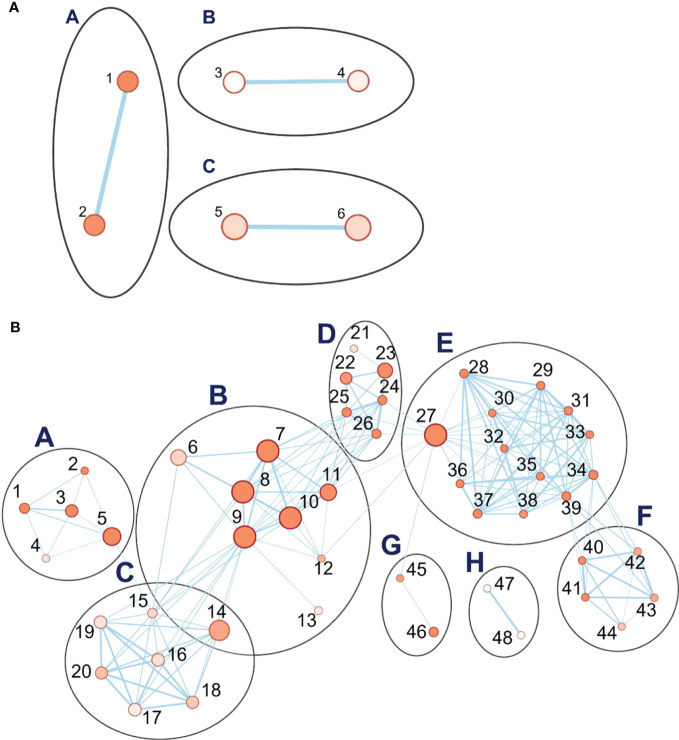
Pathway enrichment analysis of differentially expressed genes (DEGs) during the drought phase. **(A)** The set of three clusters of nodes containing upregulated genes. **(B)** Eight interlinked clusters of interlinked nodes containing genes downregulated during the drought phase. Pathway nodes are shown as small circles connected by lines (edges) if the pathways share many genes. Nodes are colored by enrichment score, and the thickness of lines according to the number of genes shared by the connected pathways. The clusters are A, photosynthesis; B, acid metabolic process; C, purine metabolic process; tRNA aminoacylation translation; E, pigment biosynthesis; F, tetraterpenoid process; G, plastid translation organization; H, plant ovary development. Nodes are numbered; descriptions for upregulated and downregulated genes are organized by node number in **Supplementary Tabless S8**, **S9**, respectively.

When drought-treated plants were compared to the well-watered controls of the same age, a simple pattern of three clusters with two nodes, each comprising the upregulated genes, was seen (**Figure 7**). Detailed information on key nodes with the clusters is presented in **Supplementary Table S8**. Cluster A, which has the highest statistical significance and contains 113 genes, is annotated as “response to water” (GO:0009415) and to “acid chemical” (GO:0001101), meaning any process that responds to the availability of water (e.g., under drought), or to an anion. In this cluster, the dehydrin genes have the highest differential expression, ranging from 100- to 3458-fold upregulation during drought, followed by the late-embryogenesis-abundant (LEA) protein Lea14 at 60-fold (**Supplementary Table S12**). Both are associated with physiological drought; dehydrin is a drought-inducible LEA II protein (Kosová et al., 2014). Conversely, three dehydrins are among the top 20 downregulated DEGs in recovery vs. drought (**Supplementary Table S6**). Of the other two clusters, one (cluster B) comprises nodes for response to salt stress (GO:0009651) and osmotic stress (GO:0006970), together with 126 genes (**Supplementary Table S8**). In cluster B, the most strongly upregulated genes are for a serine-threonine protein kinase (**Supplementary Table S12**), which is a family well connected to drought response (Saddhe et al., 2021) and salt stress (GO:0009651; 19-fold), and for an uncharacterized transcription factor (18-fold). Drought, salt, and osmotic stress response pathways are well-known to overlap (Buti et al., 2019). The third cluster C, with 250 genes, is associated with negative regulation of protein metabolic processes in two nodes (GO:0051248 and GO:0032269). These nodes contain multiple proteinase inhibitors, including a trypsin/amylase inhibitor such as was earlier shown to confer drought tolerance (Xiao et al., 2013), that are strongly upregulated in drought (64- to 694-fold).

#### 3.4.2 Downregulated networks under drought stress

During the drought stress, eight clusters, comprising 48 nodes, were downregulated (**Figure 7B**). All are related to processes of growth and development. The cluster with the highest significance and with the greatest number of genes (B) is related to metabolism of carboxylic acids and anions and comprises 3,600 genes. These are involved in nutrient supply, plant development, and processes including cell wall signaling, guard mother cell division, and pathogen virulence (Polko and Kieber, 2019). The key nodes in this cluster include metabolism of organic acids (GO:0006082), oxoacids (GO:0043436), cellular amino acids (GO:0006520), carboxylic acids (GO:0019752), and small molecules (GO:0044281) (**Supplementary Table S9**). The other interconnected clusters are associated with cluster A (654 genes), which concerns the regulation of photosynthesis, (e.g., photosystem genes, 2- to 5-fold downregulated): purine ribonucleotide metabolism, transfer RNA (tRNA) aminoacylation translation, pigment biosynthesis, and tetraterpenoid biosynthesis. A second set of linked clusters downregulated under drought stress comprises plastid translation organization (cluster G, 83 genes; GO:00032544 and GO:0009657) and pigment, including chlorophyll and carotenoid, biosynthesis (cluster E, 1588 genes; cluster F, 136 genes). A small cluster (H, 10 genes) unlinked to others is annotated as concerning plant ovary development (GO:0032544) although, given that leaf samples were analyzed, the function appears to be otherwise. Detailed information on the key nodes and clusters can be found in **Supplementary Tables S9**, **S13**. Hence, the downregulated pathways are manifold and generally involved in growth and development.

#### 3.4.3 Upregulated networks during recovery

**Figure 8:**
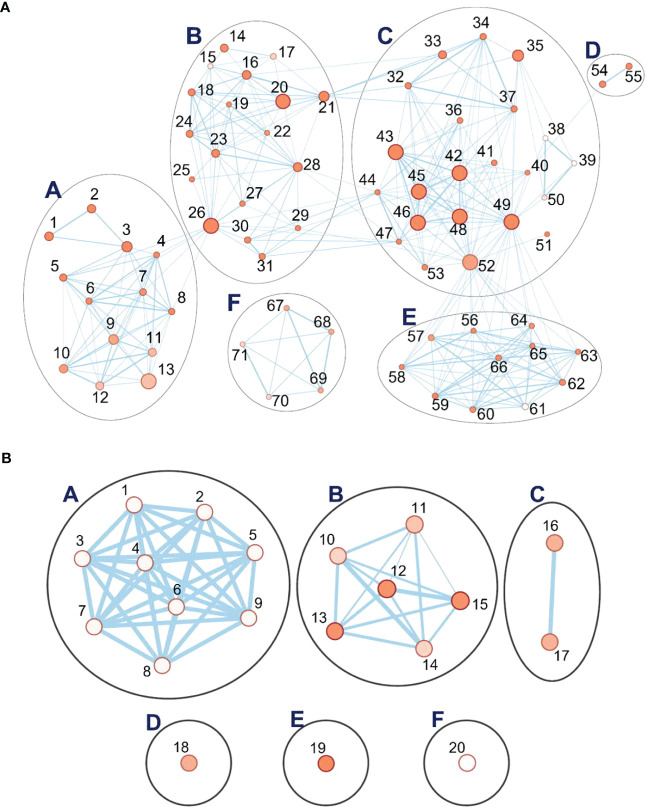
Pathway enrichment analysis of differentially expressed genes (DEGs) during the recovery phase. **(A)** Six interlinked clusters of interlinked nodes containing upregulated genes. **(B)** Six clusters of nodes containing downregulated genes. Pathway nodes are shown as small circles connected by lines (edges) if the pathways share many genes. Nodes are colored by enrichment score, and the thickness of lines according to the number of genes shared by the connected pathways. The clusters are A, positive regulation process; B, autophagy; C, response to sucrose and disaccharide; D, protein K63; E, protein neddylation; F, positive regulation of organelle. Pathway nodes are shown as small circles connected by lines (edges) if the pathways share many genes. Nodes are colored by enrichment score, and the thickness of lines according to the number of genes shared by the connected pathways. Nodes are numbered; descriptions for upregulated and downregulated genes are organized by node number in **Supplementary Tables S10**, **S11**, respectively.

Recovery is the process of restoring the physiological and molecular functions from drought-induced damage (Chen et al., 2021). As described above, gene networks related to the growth and development were downregulated during drought. In turn, during recovery, many associated with growth, plastidial function, and energy metabolism were upregulated, organized in five linked clusters of interconnected nodes (**Figure 8A**). Cluster A has 2597 genes and concerns cell wall metabolism (**Supplementary Table S10**). Its nodes include those for carbohydrate (GO:0005975), polysaccharide (GO:0005976), glucan (GO:0006073), and xyloglucan (GO:0010411) metabolism, “cell wall organization or biogenesis”, “external encapsulation structure organization” (GO:0045229), and hemicellulose metabolism (GO:0010410). Genes for biosynthesis of xyloglucan, an abundant component of the primary cell wall, were particularly strongly induced, up to 239-fold over the control plants that did not experience drought. Two of the clusters (B, 2522 genes; C, 7842 genes) include nodes associated with protein biosynthesis that were downregulated during drought but are upregulated during recovery. These include nodes for ribosome biogenesis (GO:0042254), ncRNA metabolism (GO:0034660), ribosomal RNA (rRNA) processing (GO:0006364), ribonucleoprotein complex assembly (GO:0022618), maturation of large subunit rRNA (GO:0000463), translation (GO:0006412), peptide biosynthesis and metabolism (GO:0043043 and GO:0006518), amide biosynthesis (GO:0043604), and tRNA aminoacylation and metabolism (GO:0043039 and GO:0006399). Consistent with reactivation of leaf function, plastid and chloroplast organization processes (GO:0009657 and GO:0009658) were upregulated. One unlinked cluster, for “quinone process” (GO:1901663), nevertheless, also indicates upregulation of photosynthetic function and growth, as plastoquinone and ubiquinone serve in the electron transport chains, respectively, of photosynthesis and aerobic respiration. Descriptions of the clusters and their nodes are found in **Supplementary Tables S10**, **S14**.

#### 3.4.4 Downregulated networks during recovery

The networks of genes downregulated during recovery, when compared to the controls, form six clusters (**Figure 8B**). Cluster F (13 genes in one node) is associated with the de-repression of genes associated with organelle organization (GO:0010638). Cluster A has nine nodes, each with a single gene, for processes spanning cell wall macromolecules (GO:0010981), carbohydrate metabolism (GO:0010676), trichome papilla formation (GO:1905499), among others. Descriptions of the clusters and their nodes are presented in **Supplementary Tables S11** and **S15**.

Of the other five clusters of genes downregulated during recovery, notably, three (B, D, and E) contain altogether 117 networked autophagy-related genes (ATGs) involved in ubiquitin-mediated autophagy (**Figure 8B**). The largest cluster (B) has six nodes, including ones with genes concerned with autophagosome organization and assembly. Two unlinked ATG clusters (D and E) consist of a single node each. One is for genes involved in protein K63- linked de-ubiquitination (GO:0070536). In this cluster, the genes for deubiquitinating enzyme AMSH1 are also observed. The other cluster with its single node concerns genes involved in protein neddylation (GO:0045116), the process by which the ubiquitin-like protein NEDD8 is conjugated to its target proteins in a manner analogous to ubiquitination.

The reduction in expression of the ATGs genes is modest, compared with the controls, around two-fold at most. However, of the six gene clusters with reduced expression during recovery, the top three most strongly reduced are all for ATGs genes. The *p-*values of the ATG nodes are all highly significant (mean of 0.01). Among the ATGs, the most strongly downregulated are autophagy-related protein 101 (−1.76X), beclin 1 (−1.7X), and autophagy-related protein 3 (−1.48X), all in cluster B (autophagy); a 26S proteasome regulatory subunit (−1.98X), in cluster D (protein K63-linked deubiquitination); and ubiquitin-conjugating enzyme E2 (−1.57X), in cluster E (neddylation). When gene expression levels at recovery are compared to that at drought, a similar picture emerges for ATGs (**Supplementary Table S15**). While only a few are significant at padj = 0.05, most show the same trends.

### 3.5 *BARE* response during drought and recovery

For all four varieties during drought and recovery (**Table 5**, **Supplementary S16**), the mRNA levels matching the *BARE gag* region was analyzed by qPCR; in GP, additionally, the abundance of mRNA containing the 5′ untranslated leader (UTL) regions corresponding to expression from TATA1 and TATA2 (Chang and Schulman, 2008), respectively, for the genomic RNA (gRNA) and mRNA, was measured (**Table 5**). As an indicator of general stress response, *hsp17* expression was examined; putative *hsp17* and *hsp21* were among the most strongly downregulated genes in recovery vs. drought (**Supplementary Table S6**).

**Table 5:** Relative expression of *hsp17*and BARE transcripts in Golden Promise during the drought and recovery phases along with TATA1- and TATA2-driven mRNA levels.

DROUGHT				
Samples	hsp17 **	gag**	TATA1**	TATA2**
Control 1	1.50 ± 0.08	0.66 ± 0.17	0.49 ± 0.09	0.60 ± 0.11
Control 2	2.75 ± 0.38	1.19 ± 0.09	0.83 ± 0.05	0.40 ± 0.01
Drought 1	8.01 ± 1.96	4.78 ± 0.30	5.15 ± 0.82	1.78 ± 0.24
Drought 2	4.06 ± 0.10	3.52 ± 0.79	2.12 ± 0.20	0.87 ± 0.19
RECOVERY				
Samples	*hsp17*	*gag*	TATA1*	TATA2
Control 1	0.64 ± 0.03	1.17 ± 0.07	0.57 ± 0.08	1.03 ± 0.13
Control 2	0.39 ± 0.09	0.35 ± 0.14	1.33 ± 0.12	0.59 ± 0.04
Recovery 1	0.38 ± 0.05	0.36 ± 0.11	0.23 ± 0.01	0.97 ± 0.08
Recovery 2	0.37 ± 0.02	0.73 ± 0.11	0.61 ± 0.07	1.06 ± 0.21

Values are given as means ± SD of three technical replicates for each plant. The asterisks indicate the significance level of *p< 0.05 and **p< 0.005 analyzed using a non-parametric Mann–Whitney U-test.

Drought-induced expression of both *hsp17* and of *BARE* (*gag*) was significantly higher (**Supplementary Tables S17**, **S18**) in GP and Arvo over the well-watered control (**Table 5**, **Supplementary S16**). During recovery, *BARE gag* and *hsp17* were downregulated in Arvo and GP (down 2.2X, Arvo; 1.3X, GP), but not in H673 (up 2.9X over control), suggesting delayed recovery in H673 (**Table 5**, **Supplementary S16**). Notably, in Morex, *gag* levels but not *hsp17* showed an inverse pattern from the other lines: decreasing 1.8-fold under drought but increasing 1.7-fold during recovery (**Supplementary Table S16**). We additionally followed the expression of the 5′ UTLs corresponding to both the gRNA (TATA1) and the mRNA (TATA2) of *BARE* in GP. TATA1 was more strongly upregulated than TATA2 under drought (**Table 5**, **Supplementary S17**). During recovery, TATA1 and HSP17 expression were below that of the well-watered control, whereas TATA2 was near to control levels. On the protein level (**Supplementary Figure S5**), *BARE* Pol, produced from the TATA2 mRNA, showed a strong drought response, which decreased but was still above the control during recovery.

## 4 Discussion

Drought tolerance and resilience are important traits for wild plants and commonly sought for crops. For crops, in contrast to wild species, not only the production of viable seed but also high yield is desirable. Hence, trade-offs between drought tolerance and concomitant stomatal water loss and CO_2_ uptake are relevant to breeding. Drought under field conditions is complex, affected by many factors: ambient heat, humidity, VPD, and their diurnal fluctuations; soil structure and hydration profile; soil and root microbiomes; the length and completeness of rain cessation (Scharwies and Dinneny, 2019). Likewise, experimental droughts in greenhouses and growth chambers can differ greatly from each other and from those in the field. Here, we sought to correlate transcriptional to physiological responses in barley on a precision feedback lysimeter platform with well-controlled soil and watering as well as continuous data collection. We recorded VPD and PAR continuously throughout the experiment, as well as the DT and increase in plant weight. The platform itself was in a greenhouse that closely reflected natural external environmental conditions beyond.

### 4.1 Physiological response of four barley varieties to drought and rewatering

We analyzed the drought and recovery responses of four barley varieties for which pilot experiments showed a difference in transpiration rates and ϴ_crit_. We have not tracked leaf water potential but rather the stomatal response to SWC; we use the terms anisohydric and isohydric as equivalent to ϴ_crit_ at comparatively low and high SWC, respectively. The SWC at which drought response is invoked is genotype-dependent; the two strategies can be found within genotypes of the same species such as barley or wheat (*Triticum aestivum *L.), which have both spring- and autumn-sown types that thereby experience contrasting rainfall patterns (Tao et al., 2017) depending on the cultivation region (Galle et al., 2013).

Morex, having the highest DT rate, reached ϴ_crit_ at VWC 0.46 cm^3^/cm^3^ on day 36, whereas GP reached ϴ_crit_ at the lowest VWC, 0.42 cm^3^/cm^3^, already on day 33 and continued to dehydrate at the fastest rate, but with the lowest initial transpiration rate. Hence, the rate of water loss after ϴ_crit_ was not linked to the initial transpiration rate, as GP dried the fastest and Arvo the slowest. Our interpretation is that GP is therefore the most anisohydric of the four. As bred in the UK, GP may be able to manage the risk as SWC falls because of its low transpiration rate and the relatively balanced precipitation throughout the growing season. Morex also reaches ϴ_crit_ at a comparatively low VWC but, nevertheless, maintains weight as well as H673, which has ϴ_crit_ at the highest VWC, so would appear to manage water loss well. Arvo appears to be the most isohydric; it, like H673, responds at a high ϴ_crit_ and maintains its weight best during drought. This is consistent with Arvo and H673 being varieties from Finland, where droughts occur early in the growing season and can persist for weeks, but where the probability of precipitation increases as the season progresses (Tao et al., 2017). All lines responded to rewatering by increasing specific weight gain (g/g), GP being the lowest, but none returned to control rates during the three days that recovery was followed. Full recovery may require longer.

### 4.2 Gene networks and differential expression in Golden Promise during drought and recovery

We focused gene expression analyses on GP, as the standard line for transformation and editing. *Drought*: Networks of genes responding to drought, salt, and acid, as well as negative regulators of metabolism, were upregulated following ϴ_crit_. Among individual genes, *glutathione s-transferase* (*GST*) was most upregulated (3 × 106-fold) over the control; at the sampling point early in recovery, it had dropped by 8.6-fold relative to drought samples. GST expression is associated with drought tolerance in barley (Rezaei et al., 2013); it scavenges drought-produced ROS for detoxification (Guo et al., 2009). Likewise, increased GST expression was observed under cold and osmotic stress in a potato genotype tolerant to these (Guo et al., 2009). The second most highly upregulated (447X) gene is chloroplastic linalool synthase (XP_044955412), a terpenoid biosynthetic enzyme; its expression fell 70-fold in recovery relative to drought. In rice, the bHLH family transcription factor *RERJ1* is drought-induced, leading to jasmonate (JA) biosynthesis and induction of linalool synthase (Valea et al., 2022). In barley, 98 among 135 detected phenolic and terpenoid compounds (linolool was not investigated) were shown to change in expression as result of drought stress (Piasecka et al., 2017). Our observed induction of linolool synthase here is consistent with a role in ABA/JA-mediated stomatal closure as reported for Arabidopsis (Munemasa et al., 2019). *Dehydrin *(*Dhn*) transcripts were also highly upregulated, 3500X over control, and among the most strongly downregulated in recovery vs. drought. Dehydrins, along with other late embryogenesis abundant (LEA) proteins such as LEA14 (also upregulated here), protect enzymes from dehydration and other environmental stresses (Liu et al., 2017). Differential *Dhn* expression was shown in cultivated and wild (*H. spontaneum*) barley to be correlated with drought tolerance (Suprunova et al., 2004; Kosová et al., 2014), likewise in *Brachypodium distachyon* (Decena et al., 2021), wheat (Brini et al., 2007; Wang et al., 2014), and rice (Verma et al., 2017).

Networks for photosynthetic and plastidial processes, metabolism, and ovary development were downregulated during drought, consistent with earlier reports for some individual genes from these processes: ribulose 1,5-bisphosphate carboxylase small subunit (*rbcS*), chlorophyll a/b-binding protein (*cab*), and components of photosystems I and II (Seki et al., 2002; Narusaka et al., 2004). Here, the most strongly downregulated gene was for cytochrome P450 (*CYP*), which conversely was the third most strongly upregulated in recovery vs. drought. The *CYP* genes are widely distributed in plants and animals (Shiota et al., 2000), forming a large family, 45 in wheat (Li and Wei, 2020), playing multiple roles (McKinnon et al., 2008), including in biotic and abiotic stress response (Li and Wei, 2020). In rice, 83 *OsCYPs* are differentially expressed, of which six are strongly induced and three are strongly downregulated, during drought (Wei and Chen, 2018).

Two genes for cortical cell delineating protein were highly (−705X and −621X) repressed during drought; while associated with roots, they appear to have a role in cell division and elongation (Isayenkov et al., 2020). These, too, had their expression strongly upregulated during recovery vs. drought. Downregulated almost as strongly (−442X) was a gene of the *bHLH* family. This widespread family of transcription factors has been shown in various plants, including wheat, to be integrated into ABA- and JA-mediated signaling, ROS scavenging, and stomatal closure, as well as to play multiple roles in development (Yang et al., 2016; Guo et al., 2021). Given that *bHLH* is strongly induced by drought and ABA, the pattern seen here suggests that induction occurs before ϴ_crit_ and that its downregulation thereafter may be linked to suppression of developmental processes.

*Recovery: *During the recovery phase, gene networks connected to growth and to chloroplast development were upregulated; these processes were downregulated under drought. The putative *PP7*, here the most upregulated individual gene vs. control during recovery, has shown to be highly expressed in Arabidopsis stomata (Andreeva et al., 1999), to regulate phytochrome signaling (Andreeva et al., 1999) and to be involved also in Arabidopsis chloroplast development (Xu et al., 2019), a function that would be consonant with recovery in the barley system.

Notably, autophagy processes were downregulated. Autophagy mechanisms are conserved in eukaryotes for turning over unwanted cytoplasmic components and maintaining homeostasis during stress (Marshall and Vierstra, 2018; Bao, 2020; Tang and Bassham, 2022). Mutant or silenced individual autophagy genes (*atg5,ATG6, atg7*, *ATG8d,*and *ATG18h*), variously in Arabidopsis, tomato, and wheat (Liu et al., 2009; Marshall and Vierstra, 2018; Zhu et al., 2018; Li et al., 2019), reduced drought tolerance. In barley, a mutant of autophagosome formation gene *ATG6* (*Beclin 1*) was upregulated under various abiotic stresses including drought, whereas its knockdown resulted in yellowing leaves in dark and H_2_O_2_ treatments (Zeng et al., 2017). We confirmed that *ATG6* is identical to 3HG0280440.1 (Morex assembly vers3) and saw a 1.47X reduction in recovery vs. drought (adjusted *p*= 0.01). While these studies indicated the importance of individual autophagy components in mediating abiotic stress response including drought, the autophagy network as a whole had not been examined. During recovery, along with *ATG6*, we observed a downregulation of many other *ATG*s in nodes connected either to initiation of autophagy, vesicle nucleation, expansion, or autophagosome formation. The likely importance of the suppression of autophagy during recovery is indicated by COST1 (constitutively stressed 1), attenuating autophagy in Arabidopsis under optimal growth conditions; *cost1* mutants are highly drought tolerant but very small, growing slowly (Bao et al., 2020). Of the two *COST1* matches in the barley genome, expression of HORVU.MOREX.r2.4HG0296600.1 was not seen; for HORVU.MOREX.r2.6HG0480550, expression was almost identical in drought and recovery samples and regarding the controls.

### 4.3 Response of retrotransposon *BARE* to drought and recovery

*BARE*, of which the reference genome contains 20,258 full-length copies and 6,216 solo LTRs (Mascher et al., 2021), carries out the various stages of the RLX lifecycle: It is transcribed, translated, and forms virus-like particles (Jääskeläinen et al., 2013). While the *BARE* LTR promoter was earlier shown to carry ABREs (Suoniemi et al., 1996), the capsid protein Gag to be more strongly expressed under drought (Jääskeläinen et al., 2013), and *BARE* copy number variations and insertional polymorphism patterns to be consistent with long-term drought activation (Kalendar et al., 2000), *BARE* transcription has not earlier been linked to physiologically characterized drought. Here, we found that *BARE *transcription, as measured by qPCR from the *gag* coding region, increased following ϴ_crit_ in GP, Arvo, and H673, as did the gene for heat-shock protein *HSP17,* a marker for drought stress (Guo et al., 2009); Morex, however, showed *HSP17* response but no increased *BARE* transcription. For Arvo and GP, both the *BARE gag* and *HSP17* were strongly lower in the samples taken after rewatering, whereas H673 had apparently not entered recovery, given that the *HSP17* level was still elevated. Divergent *BARE* levels in different varieties are consistent with those seen earlier (Jääskeläinen et al., 2013), where GP showed a stronger response on the Gag protein level than did cv. Bomi. For GP, we also examined the drought response for the two transcripts, one driven by TATA1, which produces the gRNA that will be reverse transcribed, and another by TATA2, which produces the translatable mRNA. In drought, TATA1 was more strongly induced than TATA2, whereas, during recovery, TATA1 showed lower expression than in control samples, perhaps indicating post-transcriptional silencing of the *BARE* gRNA. Consonant with the qPCR results, the most strongly downregulated gene in the recovery RNA-seq (HORVU.MOREX.r2.7HG0531380) matches best a superfamily *Copia* integrase domain (KAE8773625.1; 65% similar residues, *E*-value 7 × 10^-101^), likely from the *Hopscotch* family.

## 5 Conclusions

Using a precision phenotyping platform, we have identified barley varieties whose transpiration rates drop (ϴ_crit_) at different points as soil dries during drought, which may mirror adaptation to pre-flowering vs. terminal drought or to droughts of varying length. The timing of droughts regarding phenology is expected to shift across Europe over the coming decades (Appiah et al., 2023). Using ϴ_crit_ as a guidepost, we carried out a global analysis of gene expression in the transformable variety GP in response to drought and rewatering and placed the DEGs into the context of biological pathways. We identified several strongly DEGs not earlier associated with drought response in barley, including for linalool synthase, cortical cell delineating protein, and long-form PP7. These data will serve to establish candidate genes from phenotyped mapping populations and diversity sets and for analysis of the many drought tolerance QTLs heretofore identified (Zhang et al., 2017; Moualeu-Ngangue et al., 2020). We were able to confirm that the *BARE* RLX family is strongly transcriptionally upregulated by drought and downregulated during recovery unequally between cultivars and that the two *BARE* promoters are differentially drought responsive. Consistent with what may be a general epigenetic deregulation of RLXs and DNA transposons (Pontes et al., 2009; Wei et al., 2014), *Dicer-like 3* (*DCL3*) was downregulated 57-fold compared to the well-watered control during the drought phase (**Supplementary Table S3**); full analysis of transposable element expression under drought and recovery is currently in progress. In the context of recovery rate and resilience, which is key to maintenance of crop yields in the face of climate change, our observation that the autophagy gene network is downregulated during recovery is worthy of further investigation; we are currently constructing a higher resolution timeline.

## Data availability statement

The datasets presented in this study can be found in online repositories. The names of the repository/repositories and accession number(s) can be found in the article/**Supplementary Material.**

## Author contributions

MP carried out the physiological experiments and expression assays, analyzed the data, and wrote the manuscript; JT was responsible for bioinformatics analyses; MJ carried out protein expression analyses; WC contributed to qPCR analyses; AD contributed to physiological experiments; MM and AS designed the experiments; AS revised the manuscript and was project P.I. All authors contributed to the article and approved the submitted version.
