# Supplementary material for: Drought and recovery in barley: key gene networks and retrotransposon response
Source: Front Plant Sci. 2023 Jun 12;14:1193284. doi: 10.3389/fpls.2023.1193284 (PMC10291200; doi:10.3389/fpls.2023.1193284)
Supplement: Supplementary file 1 [file DataSheet_1.zip › Supplementary_Figures_1-5.PDF]

Supplementary Material

Drought and recovery in barley: key gene networks and retrotransposon response

Authors: Maitry Paul, Jaakko Tanskanen, Marko Jääskeläinen, Wei Chang, Ahan Dalal, Menachem Moshelion, Alan H. Schulman

Author for correspondence: Alan H. Schulman, *alan.schulman@helsinki.fi

The following Supporting Information is available for this article:

Supplementary Figures:

Figure S1. Daily transpiration.

Figure S2. Daily change in specific plant weight.

Fig S3. Plot of dispersion estimates for RNA-seq.

Figure S4. Principal Components Analysis (PCA) plot generated from RNA-seq data.

Figure S5. Comparison of *BARE* Pol protein levels in drought and recovery over control in GP.

Supplementary Tables (in excel file)

Table S1. Primers used in qPCR.

Table S2. Upregulated genes, drought phase.

Table S3. Downregulated genes, drought phase.

Table S4. Upregulated genes, recovery phase.

Table S5. Downregulated genes, recovery phase.

Table S6. Top 20 upregulated genes, recovery vs drought.

Table S7. Top 20 downregulated genes, recovery vs drought.

Table S8. Network analysis of upregulated genes, drought phase.

Table S9. Network analysis of downregulated genes, drought phase.

Table S10. Network analysis of upregulated genes, recovery phase.

Table S11. Network analysis of downregulated genes, recovery phase.

Table S12. Clusters and representative network nodes, upregulated genes, drought phase.

Table S13. Clusters and representative network nodes, downregulated genes, drought phase.

Table S14. Clusters and representative network nodes, upregulated genes, recovery phase.

Table S15. Clusters and representative network nodes, downregulated genes, recovery phase.

Table S16. Relative expression of *HSP17* and *BARE gag* mRNA levels.

Table S17. Mean rank by Mann Whitney U test of qPCR expression levels for GP.

Table S18. Mean rank by Mann Whitney U test of pPCR expression levels for Arvo, H673, and Morex.

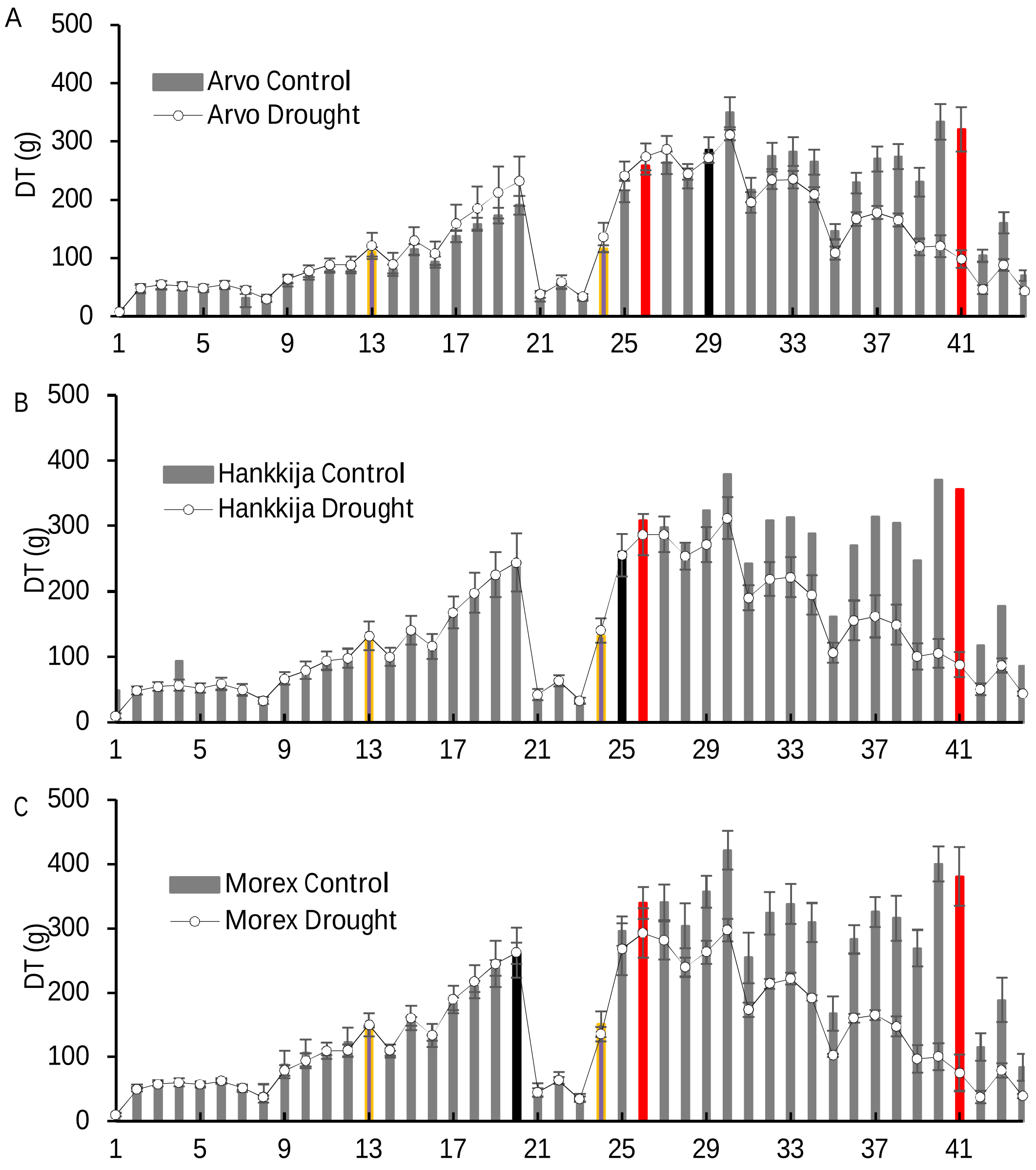

Figure S1

Daily transpiration. (A) Arvo, (B) Hankkija 6673 (H6673), (C) Morex. Yellow bars represent the start and end of Dry Phase I, red bars the start and end of Dry Phase II. The black bar represents the day when the transpiration of the droughted plants diverged from the controls.

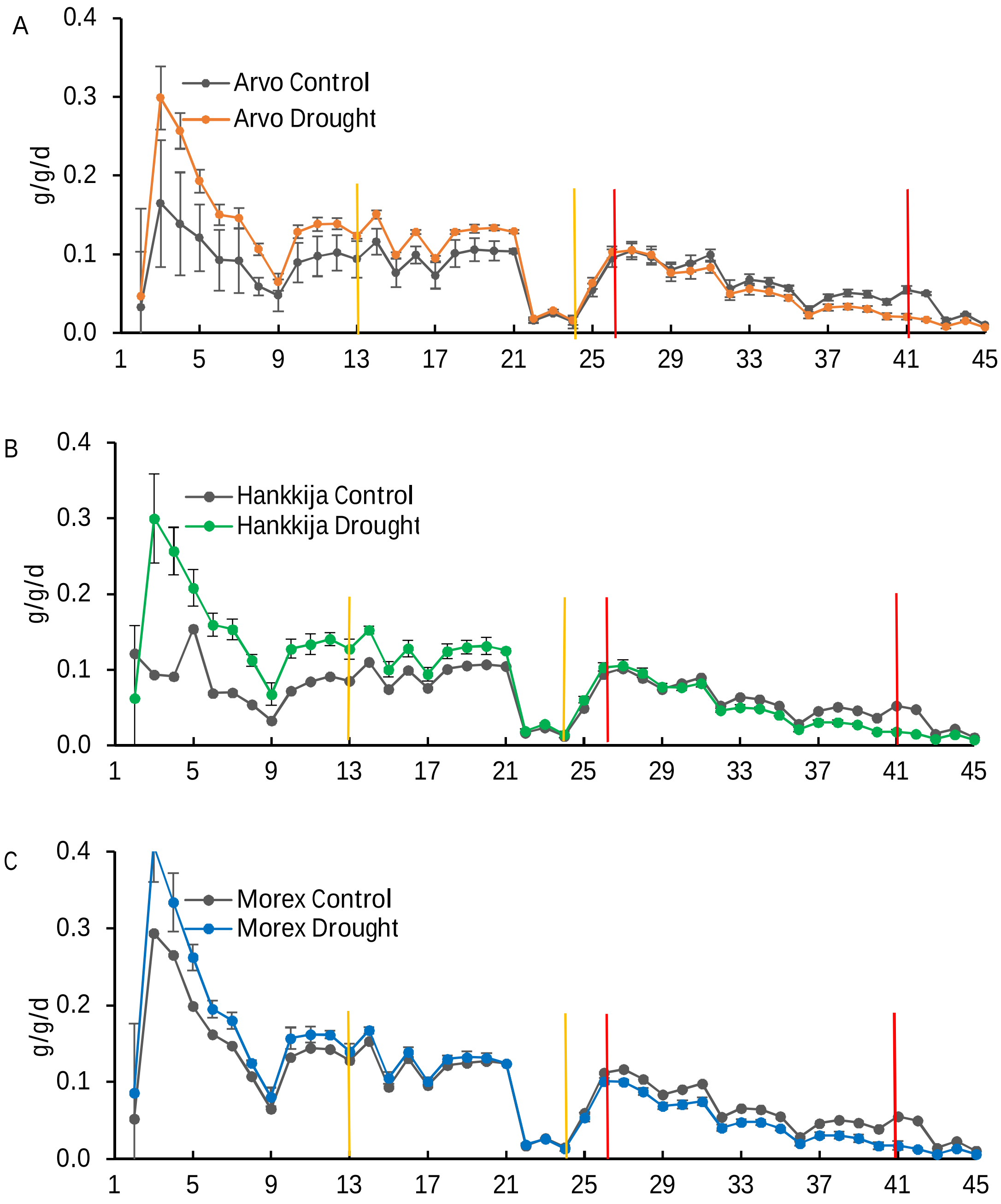

Figure S2

Daily change in specific plant weight. (A) Arvo, (B) Hankkija 673 (H673), (C) Morex. Yellow bars represent the start and end of Dry Phase I, red bars the start and end of Dry Phase II.

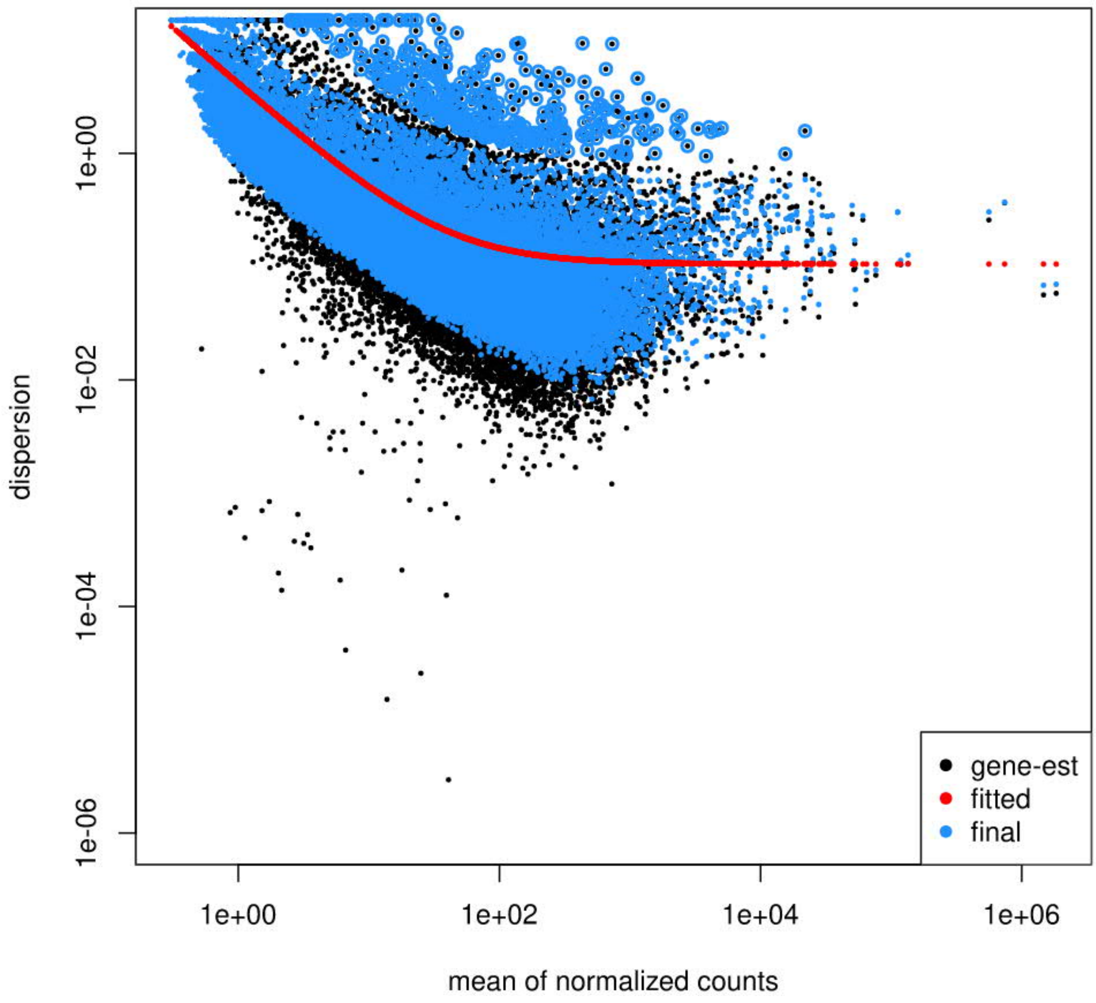

Figure S3

Plot of dispersion estimates. DESeq2 dispersion plot showing dispersion of each gene (black), the trend line for all samples (red), the corrected value of dispersion (blue), and outliers (black dot surrounded in blue) are shown.

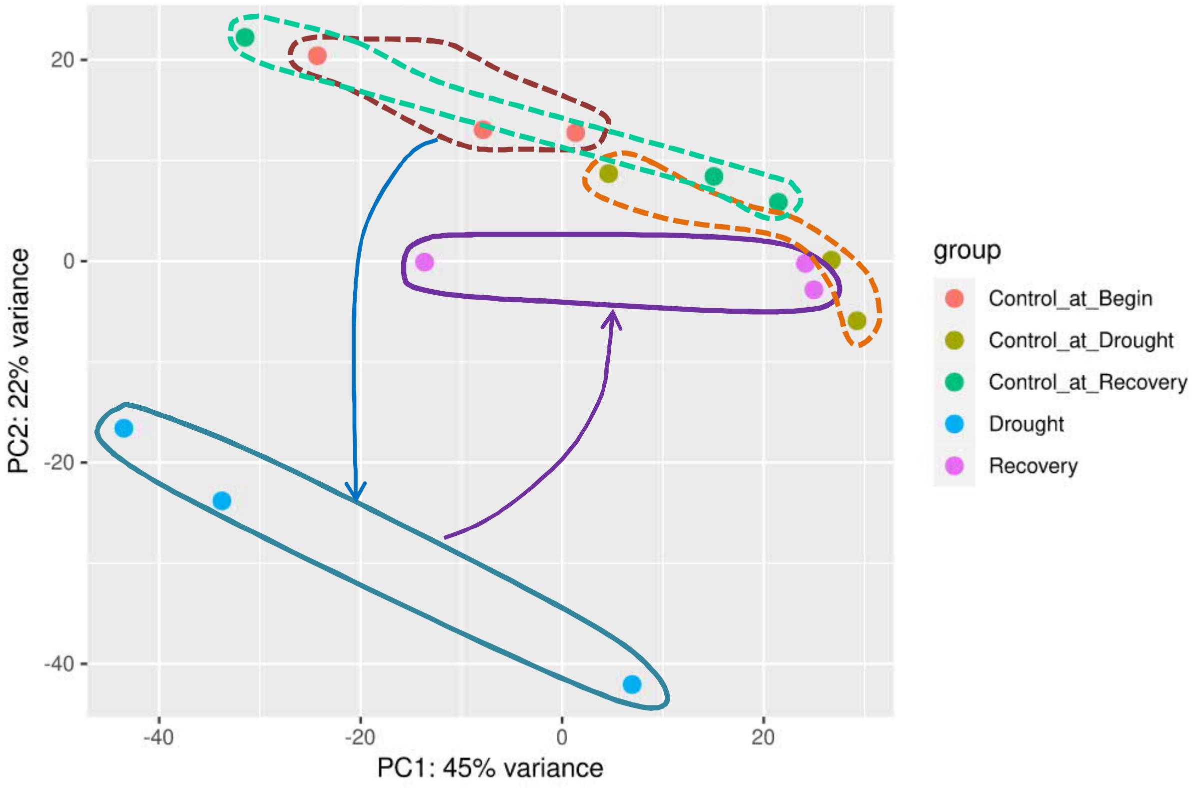

Figure S4

Principal Components Analysis (PCA) plot generated from RNA-seq data. DeSeq2 plot of the two major variance components for the groups of samples. Groups are differentiated by different colors: control at beginning (orange), control at drought (olive green), control at recovery (green), drought (blue), and recovery (purple). Samples within each group are bounded by lines in the group color, dashed for the controls. The arrows suggest the movement of gene expression pattern through two-dimensional variance space during the course of the experiment.

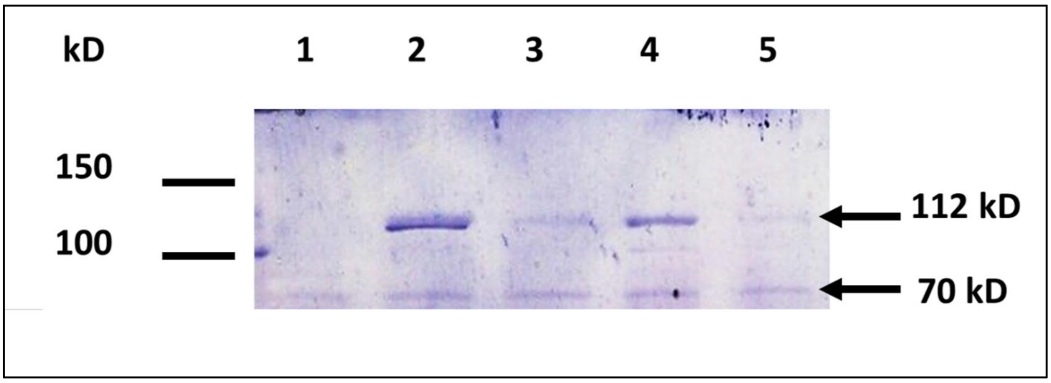

Figure S5

Comparison of BARE Pol protein levels in drought and recovery over control in GP. (1) day 12, well-watered control; (2) day 41, drought; (3) day 41, control; (4) day 44, rewatered; (5) day 44, control.
